# Routinizing the Use of Evidence in Policy – What Is Needed? Comment on "Sustaining Knowledge Translation Practices: A Critical Interpretive Synthesis"

**DOI:** 10.34172/ijhpm.2023.7604

**Published:** 2023-03-13

**Authors:** Tanja Kuchenmüller, Laura dos Santos Boeira

**Affiliations:** ^1^Research for Health, Science Division, World Health Organization, Geneva, Switzerland; ^2^Instituto Veredas, São Paulo, Brazil

**Keywords:** Knowledge Translation, Evidence-Informed Policy, Institutionalization, Institutional Capacity, Sustainability, Conceptual Framework

## Abstract

In their study of sustaining knowledge translation (KT) practices, Borst et al found that this process is an interplay of: (*i*) constructing and extending networks, (*ii*) creating contexts that support KT practices, and (*iii*) understanding how actors create, maintain, and disrupt institutions. Their article is an important contribution to the body of research promoting KT. In this commentary we reflect on the convergences and differences between the concepts of ‘sustaining’ and ‘institutionalizing’ KT, highlighting domains and processes related to the institutionalization, providing an analysis of KT landscape in Brazil and making a case for the need to increase countries’ routine use of evidence.

 The unprecedented evidence climate presented by the COVID-19 pandemic provides a unique window to promote knowledge translation (KT) and evidence-informed health policy-making (EIHPM).^1^ Many lessons related to the mobilization and translation of evidence have been learned that we need to capitalize and leverage on.^2,3^ In particular the need for sustainable KT structures and processes has been vividly brought to the fore to support national policy processes. Country-owned KT infrastructures and mechanisms are key to implement an equitable COVID-19 recovery process, increase country resilience against the current and future crises, and address societal challenges in ‘peace’ times. It has become clear, though, that capacities, productive relation and partnerships between researchers, policy-makers and other stakeholders, just as trusted mechanisms, need time to be in place before a crisis hits.^1,4^

 Despite the increased international recognition and efforts to promote the systematic and transparent use of evidence in policy and practice, KT initiatives continue to be frequently linked to externally funded, time-bound projects disconnected from local needs and ownership. As a consequence, KT practices are susceptible to being discontinued once projects come to an end, leading to an investment loss for the organizations involved and jeopardizing the achievement of set public health goals.^5,6^

 Realizing the need to identify means by which countries are guided in maintaining their KT work, Borst et al conducted a critical interpretive synthesis, exploring both the meaning and processes of sustaining evidence-to-policy activities. The resulting conceptual framework is a valuable contribution to the body of research literature: three main processes were identified as constituting the sustainment KT process:* translating, contexting, and institutionalizing.*^5^

 Departing from the same line of reasoning as Borst and colleagues, the World Health Organization (WHO) has recently developed an institutionalization checklist for its Member States relying on domains and processes for institutionalizing EIHPM.^6,7^ For years, WHO with its Evidence-informed Policy Network^[1]^ (EVIPNet) has been promoting Member States’ capacity in KT and EIHPM by providing training workshops and developing, jointly with its partners, cutting-edge KT approaches and tools to empower countries and strengthen their leadership in formulating and implementing health policies embedded in sound evidence. In 2021, to accelerate processes towards more mature KT institutionalization, EVIPNet developed a new global roadmap for supporting evidence into policy and action. It was launched in the form of a call for action at the WHO Evidence-to-Policy Summit.^8^

 Before that, beyond supporting the conducting of an EVIPNet situation analysis^9^ to assess the country’s KT landscape and identify the institutional niche for and mechanisms to establish national knowledge translation platforms (KTPs)^[2]^, no concrete Guidance was available to countries for the implementation of more strategic and comprehensive KT institutionalization processes towards better health policies. Both the article and the checklist contribute greatly to this: while Borst and colleagues outline conceptually the (re)creation of KT systems, the WHO approach provides clear steps towards its institutionalization, aiming that governments can indeed routinize the use of evidence in health policy-making.

 In the following we will briefly describe how the work of Borst et al and of WHO complement and mutually enrich each other to provide a more comprehensive view of sustaining and institutionalizing KT. We will first try to entangle the concepts of sustainment and institutionalization, secondly, outline the domains and processes identified on KT institutionalization efforts, providing the example of Brazil as a case study of how the concepts developed by Borst et al and by WHO can be associated in a country’s analysis, and finally focus on a call to action for increasing countries’ resilience against future crisis.

## Sustain and Institutionalize Knowledge Translation – Two Sides of the Same Coin?

 The conceptual separation between institutionalization and sustainability reveals itself as difficult, with the terms often used synonymously.^6^ In the context of KT, both concepts aim to promote the routinization of evidence-to-policy activities to ensure that policy processes are regularly informed by relevant knowledge while relying on strategic linkages and exchange between policy-makers, researchers and other stakeholders, and making the activities last.^5,6^ Importantly, both concepts also need to be seen as being a process, ie, a set of ongoing activities, just as an outcome. Reflecting the latter, WHO suggests defining KT institutionalization as a^7^:

 “*Process and outcome of (re-)creating, maintaining and reinforcing norms, regulations, and standard practices that, based on collective meaning and values, actions as well as endowment of resources, allow evidence to become – over time – a legitimate and taken-for-granted part of policy-making.”*^6^

 “*Institutionalization relies on building relationships and interactions between those stakeholders that produce research evidence, and how they connect, interact and network with the ones who will use this knowledge. It is also affected by institutional capacities to conduct the processes and uphold the standards.”*^10^

 Although one could argue that with its focus on longevity and duration, the concept of sustainability omits to consider how much a practice is actually built-in to a society and enshrined in its structures, rituals and beliefs, a range of authors (including Borst et al) argue that institutionalization is an important phase instrumental to achieving sustainability.^6^ In other words, while sustainability can be seen as the umbrella concept under which institutionalization is featured, sustainment and institutionalization both aim for the similar outcomes in terms of securing the permanence of activities and the related durability of societal benefits such as the achievement of the Sustainable Development Goals.

## WHO Domains and Process Framework of Knowledge Translation Institutionalization – What Is the Interlink With the Practice-Centred Approach Proposed by Borst et al?

 Despite the similarities in terms of goals and outcomes, the approaches taken forward by the work of Borst et al and WHO considerably differ. While Borst et al promote a practice-centred perspective, WHO has focused with its work on what the authors call a ‘factor’-centred approach,^5^ relying on the identification of key institutional and contextual factors contributing to institutionalizing and promoting the longevity of KT at country level. WHO supplements the ‘factor’-centred approach by providing guidance on how countries can initiate and advance their journey of KT institutionalization, including how the proposed institutionalization factors can be enhanced.^6^

 Borst and colleagues’ article and WHO share a similar definition of KT^[3]^, understood as encompassing both the transformation of knowledge and the creation of engagement processes and connections between the producers and users of research. The process of translation in Borst and colleagues’ framework goes hand-in-hand with the contexting of KT practices, meant as the day-to-day work of actors constructing contexts that are conducive to reproducing KT practices.^5^

 While WHO’s work does not explicitly feature KT practices, the domains and process frameworks point out the need for actorial efforts and the continuous, pro-active reproduction and adaption of practices to advance the institutionalization agenda. This includes the continuous reproduction and strengthening of the domains just as the routinization both of KT processes and of policy-makers regular requesting evidence, enabled by the institutionalization domains.^6^

 The domains framework, in a nutshell, combines six conceptually distinct, but interlinked domains that offer guidance to KT actors seeking to improve the institutionalization of their efforts:

 “ 1) Governance – wide range of rule-making and steering-related functions, including institutionalized structures, mandates or platforms that span the boundaries between research and policy;

 2) Standards and routinized processes – tools and protocols, as well as institutional memory and documentation processes, to ensure minimum standards and high-quality KT products and processes;

 3) Leadership and commitment – strong charismatic leadership and champions who have the ability to affect the long-lasting adoption of EIPM directly, through allocation of resources (human and material) and indirectly, through encouragement, support, and mentorship;

 4) Resources and capacity-building/strengthening – availability and development of human, financial, material and information resources. Having a critical mass of people, within and outside of the organization, skilled in applying KT routinely and consistently, and throughout time;

 5) Partnership, collective action and support – extent to which stakeholders interact in the “organizational field,” providing a mechanism for continued engagement and involvement of multiple stakeholders for the same cause, joint problem-solving, identification of resources for ongoing KT, and continued technical support; and

 6) Culture – basic values, assumptions and beliefs that are considered valid and are being disseminated and promoted as daily practices. These allow for a common understanding of what KT is, what value it can bring about and what is to be expected in terms of activities and benefits.”^6,7^

 Actorial efforts become even more pronounced in WHO’s process framework of KT institutionalization. Indeed, institutionalization, to be understood as a non-linear process comprising of the phases of pre-institutionalization, semi-institutionalization, re-institutionalization and, potentially, renewed de-institutionalization, is relying on a constant negotiation and reproduction of practices shaping the institutional landscape and contexts over time. Conscious steps need to be taken to influence the general discourse, legitimize practices, and mobilize both material and immaterial assets to effect change at the beginning of the institutionalization process, or maintain the status quo through the reproduction of social order and standardized, habitualized behaviours once a mature stage of institutionalization has been reached.^6,7^

## Practices, Domains and Processes: Reflecting on Knowledge Translation Institutionalization in Brazil

 In March 2022, members from Instituto Veredas, a Brazilian evidence center, applied the WHO checklist while presenting the national evidence ecosystem to an international community of practice. From the practice-centred approach of Borst and colleagues, the idea of KT as (re-)building of networks by creating connections between places of knowledge production and utilization resonates with the Brazilian experience, due to ongoing efforts related to the institutionalization of EVIPNet Brazil and the Brazilian Coalition for Evidence. Also, the double-meaning of ‘context,’ highlighted by Borst et al as an environment to which the KT practice needs to be attuned to and as a concrete set of facilitators and barriers, was useful to characterize how KT institutionalization changes in a country with so much local diversity such as Brazil. From the WHO domains and processes, it was possible to frame the country as having reached an institutionalization stage in the Leadership and Partnership domains. Despite having some KTPs embedded in governments, Brazil still is in the semi-institutionalization stage in the Governance, Resources and Standards domains, especially when moving away from the health sector. Finally, the COVID-19 pandemic sparked debates that fostered the Culture domain, which is still very much in the pre-institutionalization stage. From this comprehensive reflection on the national context, Instituto Veredas alongside the Brazilian Coalition for Evidence designed an early-career introductory course and mentorship on KT for researchers who are gender and race-diverse and focus on evidence for social policies to the North and Northeast regions of Brazil. This capacity-strengthening initiative was launched on June 2022 as a measure to improve the underserved domains of culture and human resources, reflecting a direct combination of the practice and factor-centred approaches to KT institutionalization.

## A Call to Sustain and Institutionalize Knowledge Translation – to Increase Country’s Routine Use of Evidence

 To scale-up KT institutionalization, case studies and a pilot testing of the WHO institutionalization checklist are required. In addition, further research is needed to fully understand the complex interrelations and system equilibrium between the six institutionalization domains as some domains may be more relevant in certain sociopolitical and cultural context than in others.^6^ We agree with Borst and colleagues’ suggestions of further empirical studies to understand what actors do to create, amend, or dismiss institutions, and how they interact and shape the KT context.^5^

 The most pressing step now it to mobilize political commitment and investments to put in place the national, regional and global implementation plans for KT institutionalization.^2^ We urge governments, intergovernmental organizations and other key stakeholders, such as bi-/multilateral donors and foundations, to join our efforts so as to the routine use of evidence in decision-making becomes the norm and a meaningful difference to national policy-making and the lives of people can be made.

## Ethical issues

 Not applicable.

## Competing interests

 TK is employed full-time with WHO, working in the fields of research, evidence and policy. LdSB had a consultancy contract with WHO on institutionalizing EIHPM.

## Authors’ contributions

 TK contributed to the conception, writing and formatting of the commentary, LdSB contributed to the writing and reviewing of the commentary. All authors read and approved the final manuscript.

## Disclaimer

 The authors alone are responsible for the views expressed in this paper and they do not necessarily represent the views, decisions or policies of the institutions with which they are affiliated.

## Endnotes


^[1]^ EVIPNet’s website: https://www.who.int/initiatives/evidence-informed-policy-network
^[2]^ According to EVIPNet, a KTP “is an organization or network that brings together the worlds of research and policy” (p. 6).^11^ A KTP develops strategies to better understand a particular policy issue; harvest, synthetize, package, disseminate and broker local and global evidence to inform policy development and implementation among stakeholders; and strengthen KT capacities of researchers, policy-makers and other stakeholders.^11^

^[3]^ WHO defines KT as “the exchange, synthesis, and effective communication of reliable and relevant research results. The focus is on promoting interaction among the producers and users of research, removing the barriers to research use, and tailoring information to different target audiences so that effective interventions are used more widely” (p. 140).^12^


## References

[1] Kuchenmüller T, Reeder JC, Reveiz L (2021). COVID-19: investing in country capacity to bridge science, policy and action. BMJ Glob Health.

[2] Kuchenmüller T, Lavis J, Kheirandish M (2022). Time for a new global roadmap for supporting evidence into action. PLOS Glob Public Health.

[3] World Health Organization (WHO). Evidence As a Catalyst for Policy and Societal Change: Towards More Equitable, Resilient and Sustainable Global Health. Geneva: WHO; 2022.

[4] El-Jardali F, Bou-Karroum L, Fadlallah R (2020). Amplifying the role of knowledge translation platforms in the COVID-19 pandemic response. Health Res Policy Syst.

[5] Borst RAJ, Wehrens R, Bal R (2022). Sustaining knowledge translation practices: a critical interpretive synthesis. Int J Health Policy Manag.

[6] Kuchenmüller T, Boeira L, Oliver S (2022). Domains and processes for institutionalizing evidence-informed health policy-making: a critical interpretive synthesis. Health Res Policy Syst.

[7] World Health Organization (WHO). Supporting the Routine Use of Evidence During the Policy-Making Process: A WHO Checklist. Geneva: WHO; 2022.

[8] World Health Organization (WHO). Together on the Road to Evidence-Informed Decision-Making for Health in the Post-Pandemic Era: New Evipnet Call for Action. Geneva: WHO; 2021.

[9] EVIPNet Europe. Situation Analysis Manual. Copenhagen: WHO Regional Office for Europe; 2017.

[10] Alwan A, Ali M, Aly E (2017). Strengthening national health information systems: challenges and response. East Mediterr Health J.

[11] EVIPNet Europe. Introduction to EVIPNet Europe: Conceptual Background and Case Studies. Copenhagen: WHO Regional Office for Europe; 2017.

[12] World Health Organization (WHO). World Report on Knowledge for Better Health: Strengthening Health Systems. Geneva: WHO; 2004.

